# Simultaneous Detection and Quantification of *Phytophthora nicotianae* and *P. cactorum*, and Distribution Analyses in Strawberry Greenhouses by Duplex Real-time PCR

**DOI:** 10.1264/jsme2.ME12177

**Published:** 2013-04-24

**Authors:** Mingzhu Li, Minoru Inada, Hideki Watanabe, Haruhisa Suga, Koji Kageyama

**Affiliations:** 1 River Basin Research Center, Gifu University, Gifu 501–1193, Japan; 2 Saga Prefectural Agricultural Research Center, Saga 840–2205, Japan; 3 Gifu Prefectural Agricultural Technology Center, Gifu 501–1152, Japan; 4 Life Science Research Center, Gifu University, Gifu 501–1193, Japan

**Keywords:** *Phytophthora nicotianae*, *P. cactorum*, internal transcribed spacer regions, ras-related protein gene *Ypt*1, TaqMan probe

## Abstract

*Phytophthora nicotianae* and *P. cactorum* cause *Phytophthora* rot of strawberry. A duplex real-time PCR technique for simultaneous detection and quantification of the two pathogens was developed. Species-specific primers for *P. nicotianae* and *P. cactorum* were designed based on the internal transcribed spacer regions (ITS) of rDNA and the ras-related protein gene *Ypt*1, respectively. TaqMan probes were labeled with FAM for *P. nicotianae* and HEX for *P. cactorum*. Specificities were demonstrated using 52 isolates, including various soil-borne pathogens. Sensitivities for *P. nicotianae* and *P. cactorum* DNAs were 10 fg and 1 pg, respectively. The technique was applied to naturally infested soil and root samples; the two pathogens were detected and the target DNA concentrations were quantified. Significant correlations of DNA quantities in roots and the surrounding soils were found. The minimum soil DNA concentration predicting the development of disease symptoms was estimated as 20 pg (g soil)^−1^. In three strawberry greenhouses examined, the target DNA concentrations ranged from 1 to 1,655 pg (g soil)^−1^ for *P. nicotianae* and from 13 to 233 pg (g soil)^−1^ for *P. cactorum*. The method proved fast and reliable, and provides a useful tool to monitor *P. nicotianae* and *P. cactorum* in plants or soils.

The Oomycete genus *Phytophthora*, which includes some of the most destructive plant pathogens, causes considerable economic losses to food crops and ornamentals (10). Species like *P. nicotianae* and *P. cactorum* have wide host ranges and infect roots, crowns and fruits, and are serious soil-borne pathogens worldwide (10, 26, 32).

In Shizuoka prefecture (Japan), an outbreak of *Phytophthora* rot of strawberry occurred in 1978 (33) and both *P. nicotianae* and *P. cactorum* were reported as the pathogens responsible (15, 33). The symptoms are very similar to those caused by the anthracnose pathogen *Colleotrichum gloeosporioides*, which is responsible for a loss of almost 350 million dollars to Japanese strawberry producers over the last four years. It is important to distinguish the diseases because the disease control strategies are different for each disease. Moreover, despite the wide host range of the two *Phytophthora* species, their distribution in Japan remains unknown; therefore, it is desirable to establish a simple and quick method to detect and quantify these pathogens.

The control of soil-borne diseases caused by *Phytophthora* spp. is often difficult due to the release into the soil of resistant perennating structures, oospores and/or chlamydospores. Early diagnosis and detection of pathogens in plants, soil and water are very important to determine their transmission modes. PCR has become the primary method of identifying plant pathogens (9, 11, 23). Diagnostic PCR methods and specific primers have been developed for *Phytophthora* species including *P. nicotianae* (12, 18, 22, 28) and *P. cactorum* (2, 5, 21, 30), but most of these studies aimed at the detection of a single pathogen. Multiplex PCR assays allow the simultaneous detection of several species, and facilitate large-scale sample processing (25); however, multiplex PCR has been applied rarely in plant pathology (13, 14, 24, 31, 34, 35). This is partially due to the difficulties related to the development of quantitative multiplex assays and to the reduced sensitivity of multiplex PCR compared with simplex PCR (31).

Real-time PCR chemicals utilized to detect phyto-pathogenic micro-organisms can be grouped into amplicon sequence-non-specific (SYBR Green) and sequence-specific methods (TaqMan, Molecular Beacons, Scorpion PCR, *etc*.) (27). SYBR green is a non-specific dye that fluoresces when intercalated into double-stranded DNA, whereas amplicon sequence-specific methods are based on the labeling of primers or probes with fluorogenic molecules that allow the detection of specific amplified target sequences (34). Real-time PCR-based techniques are faster, more sensitive, more easily automated, and do not require post-amplification procedures; therefore, these techniques have been adopted widely for the quantitative detection of fungal and oomycete plant pathogens. The quantitative detection of plant pathogens facilitates the monitoring of pathogens and the study of their distribution, enabling improved disease control and minimum usage of fungicides.

A good choice of gene for primer and probe designing is crucial for PCR-based diagnostic methods. Although the internal transcribed spacer (ITS) regions of the nuclear-encoded ribosomal RNA genes (rDNA) are widely used to identify and detect *Phytophthora* species (7), they are not always sufficiently variable to separate closely related taxa (20, 29, 31). Kong *et al.* (18, 19) reported that the elicitin gene *par*A1 and the putative storage protein genes (*Lpv*) were useful as the target for the specific detection of *P. cinnamomi* and *P. nicotianae*, respectively; however, neither gene contains introns, and neither is likely to be variable enough to distinguish a broad range of species (29). The ras-related protein gene (*Ypt*1) (6) contains sufficient variation suitable for the development of molecular markers for almost all *Phytophthora* species, without intra-specific variability (29).

The objective of this study was to develop a duplex real-time PCR technique for the simultaneous detection and quantification of *P. nicotianae* and *P. cactorum* in soils and strawberry roots. We also investigated the distribution of the two pathogens in various strawberry greenhouses. The duplex quantification of *P. nicotianae* and *P. cactorum* provided a useful tool for the diagnosis of strawberry pathogens.

## Materials and Methods

### Species and strain maintenance

Thirty-two *Phytophthora* species, eleven oomycetes (genera *Pythium* and *Saprolegnia*), and five soil-borne pathogens including *Plasmodiophora*, *Pyrenochaeta*, *Rhizoctonia*, and *Verticillium*, were used (Table 1). The *P. nicotianae* isolates with different hosts were provided by the CBS (Centraalbureau voor Schimmelcultures, Utrecht, the Netherlands) and collected from different prefectures (Gifu, Chiba and Okayama) of Japan. The *P. cactorum* isolates from strawberry were collected from different prefectures (Gifu, Chiba, Okayama and Tokushima) of Japan. Other *Phytophthora* species, *Pythium* species and fungal pathogens were collected from several scientific resource institutions and Gifu University Culture Collection. All culturable isolates were maintained on corn meal agar (CMA) or potato-dextrose agar (PDA) at 20°C in the dark.

### Collection of samples

For the survey of pathogen quantities in strawberry greenhouses, soil samples were collected in July 2011 and February 2012. In July 2011, after sterilization of the soil, 26 soil samples, including 13 from the north and 13 from the south side of the greenhouse, were collected from a strawberry greenhouse in Saga prefecture. Thirty and thirteen soil samples were collected from two strawberry greenhouses in Gifu prefecture, respectively. In February 2012, during the cultivation of strawberry, another 26 soil samples were collected in the same greenhouse in Saga. For each sample, approximately 50 g soil was collected from a depth of 5–10 cm. In addition, 15 diseased roots and attached soils were collected independently from each sampling plot in the greenhouse in Saga (Fig. 3).

### DNA extraction from mycelia and soil

Total genomic DNA from mycelia was extracted according to the procedure of Kageyama *et al.* (17). Mycelia grown on V8 juice broth medium were used for DNA extraction from culturable species. For soil DNA extraction, the method refined by Kageyama *et al.* (17) was modified by incorporating a magnetic bead purification step (MagExtractor-Plant Genome; Toyobo, Osaka, Japan) to purify soil DNA extracts as described by Li *et al.* (24). Briefly, 0.2 g soils were added to autoclaved 2 mL Eppendorf tubes containing 0.2 g glass beads of 1 mm diameter. The soil was suspended in 250 μL extraction buffer (100 mM Tris HCl [pH 9.0], 40 mM EDTA, 2% [w/v] sodium dodecyl sulfate, 0.8% [w/v] skim milk; Difco Laboratories, Detroit), and RNase A at 200 μg mL^−1^ (Nippon Gene, Toyama, Japan), and then vigorously vortexed at 4,200 rpm for 1 min. One hundred fifty milliliters of benzyl chloride was added to the mixture, and the tube was vigorously vortexed for 2 min. After 15 min of incubation at 60°C, 150 μL of 3 M sodium acetate was added to the suspension and the mixture was lightly vortexed. After 15 min of incubation on ice, the suspension was cleared by two rounds of centrifugation at 18,000×*g* for 10 min, and the upper layer was transferred to a clean tube. The extracted DNA was purified according to the manufacturer’s instructions in the purification step of the MagExtractor-Plant Genome kit. DNA was resolved in 50 μL TE buffer until the next step. For the preparation of a root sample, the root was cut into pieces of approximately 1 mm size with a sterile blade and 0.1 g was used for further experimentation.

### Primer and probe design

Specific primers for *P. nicotianae* and *P. cactorum* were designed from the alignment of the DNA sequence in the ITS region obtained from 52 *Phytophthora* and 3 *Pythium* species, and the *Ypt*1 gene obtained from 42 *Phytophthora* and 3 *Pythium* species using BioEdit ver. 7.0.0 (Isis Pharmaceuticals, Dublin, Ireland) (Table 2). All of the ITS sequences and the *Ypt*1 gene sequences were collected from the NCBI DNA database. Primers and probes were designed using the Beacon Designer Ver. 7.51 (PREMIER Biosoft International, Palo Alto, CA, USA). Specific probes were labeled with the reporter dyes FAM for *P. nicotianae* and HEX for *P. cactorum* to allow the simultaneous detection of the two pathogens in a single reaction of duplex real-time PCR. Eclipse Dark quencher (Epoch Biosciences, Bothell, WA, USA), a non-fluorescent dye with a maximum absorption at 522 nm, quenches effectively a broad group of fluorescent dyes with emissions of 390–625 nm in dual labeled probes, and was used in this study.

### Amplification conditions

Conventional PCR reactions were performed in a total volume of 25 μL containing 1 μM of the developed primers, 1 unit FastStart *Taq* DNA polymerase (Roche Applied Science, Mannheim, Germany), 0.2 mM dNTP mixture, 1×PCR buffer (10 mM Tris-HCl, pH 8.3, 50 mM KCl, and 1.5 mM MgCl_2_), 10 ng bovine serum albumin (Sigma, St Louis, MO, USA) and about 50 ng DNA template. PCR amplification conditions were one cycle of 95°C for 5 min; 35 cycles of 94°C for 30 s, 62°C for 30 s, and 72°C for 1 min; and a final cycle of 72°C for 10 min. Amplicons were analyzed by electrophoresis in a 2% agarose S (Nippon Gene) gel containing GelRed (Biotium, Hayward, CA, USA) in TAE buffer and were visualized under UV light.

All real-time PCR reactions were performed in a total volume of 20 μL containing 1 μL genomic DNA solution, 1×Premix Ex Taq (Takara, Otsu, Japan), 1×ROX Reference Dye II, 4 mM MgCl_2_, and 0.8 μM of each primer for *P. cactorum*, 0.2 μM of each primer for *P. nicotianae*, and 0.2 μM of each probe. PCR amplification was programmed with one cycle of one cycle of denaturation at 95°C for 10 s and 40 cycles of 95°C for 5 s and 62°C for 34 s. Fluorescence was monitored in each PCR cycle during the annealing–extension phase at 62°C. Amplifications were performed using an Applied Biosystems StepOnePlus Real Time PCR System (Life Technologies Japan) and data acquisition and analysis were realized using the supplied StepOne software version 2.2.2 according to the manufacturer’s instructions. The cycle threshold (*C*_t_) values for each reaction were calculated automatically using StepOne software by determining the PCR cycle number at which the reporter fluorescence exceeded the background.

### Accession numbers of the sequences used in GenBank

The accession numbers of the sequences of the ITS region used in this study (Table 2) were FJ801769, FJ801963, FJ801542, AB367364, AB367366, AB367365, GU259292, AY241924, FJ801954, GU259025, GU259193, AB437135, GU594781, GU259136, FJ801913, FJ801888, GU258989, AF228099, FJ801387, AF228082, DQ275190, AF266762, EU080072, FJ801946, AY302164, FJ801470, AF266789, AF271222, FJ802005, FJ801323, L41378, GU259517, AY940661, FJ802093, GU259180, GU259090, FJ802010, GU258789, FJ801540, FJ801253, FJ802098, DQ988192, FJ801359, FJ801246, AY659739, FJ802106, FJ801904, AB511828, FJ801438, AF266774, FJ801435, AY853200, FJ801362, AB367498, FJ801828, AF266775, AB108025, AF271230, and AY598713.

The accession numbers of the sequences of the *Ypt*1 gene used in this study (Table 2) were DQ162981, HQ849999, DQ162960, HQ850000, HQ850001, DQ162953, DQ162956, DQ162972, DQ162959, DQ162971, DQ162973, HQ850002, DQ162987, DQ162989, DQ162988, DQ162952, DQ162950, HQ850003, HQ850004, DQ162963, HQ850005, DQ162974, DQ162985, HQ850006, HQ850007, DQ162980, DQ162975, DQ162991, DQ162990, HQ850008, DQ162986, EF649778, HQ850009, HQ850010, DQ162965, HQ850011, HQ850012, DQ162957, DQ162967, HQ850013, DQ162964, DQ162979, DQ162992, DQ162958, HQ850014, HQ850015, HQ850016, and HQ850017.

## Results

### Primer and probe design for real-time PCR

New specific primers for real-time PCR were designed based on the alignments of the ITS region and the *Ypt*1 gene sequences for *P. nicotianae* and *P. cactorum*, respectively. Its-nicF1 and Its-nicR3 for *P. nicotianae* were designed with an amplicon size of 312 bp, while Ypt-cacF3 and Ypt-cacR3 for *P. cactorum* were designed with an amplicon size of 122 bp (Table 3). TaqMan probes, P-nic4 and P-cac4, were selected and marked by FAM and HEX, respectively (Table 3). Tm values of primers and probes were calculated using the nearest-neighbor algorithm.

### Specificity tests in conventional PCR and real-time PCR

In conventional PCR, seven isolates of *P. nicotianae* and *P. cactorum* from different hosts and geographic locations in Japan were used together with 45 non-target species (Table 1) to test the specificity of the designed primers for each species. The presence of the extracted DNA was confirmed using a universal primer set (18S-69F and 18S-1118R) (1). The primers Its-nicF1 and Its-nicR3 only amplified the *P. nicotianae* sequences with a specific band of 312 bp, and Ypt-cacF3 and Ypt-cacR3 exclusively amplified the *P. cactorum* sequences with a unique band of 122 bp. The two target bands were clearly distinguished on electrophoresis.

To verify the specificity of the designed primers and TaqMan probes by real-time PCR, three *P. nicotianae* isolates, three *P. cactorum* isolates, and eleven closely related *Phytophthora* species belonging to Clade 1 were tested according to Blair *et al.* (3). The fluorescence of FAM increased only in samples containing *P. nicotianae* DNA, while the fluorescence of HEX increased only in those containing *P. cactorum* DNA. In other non-target samples, signals remained below the background level (Table 1).

### Optimization of real-time PCR

In order to optimize the real-time PCR procedure for *P. nicotianae* and *P. cactorum*, various concentrations of primers and probes were tested (0.1, 0.2, 0.4, and 0.8 μM). Among the sixteen concentration combinations of primers and probes, 0.8 μM primers with 0.2 μM probe for *P. cactorum*, and 0.2 μM of primers as well as probe for *P. nicotianae* were found to offer the fastest and most stable amplifications. Annealing temperatures of 58, 60 and 62°C were tested, and the amplification started fastest at 62°C. The concentration of magnesium proved to be an important factor in the duplex real-time PCR, and 4 mM magnesium chloride was found to support the multi-amplification best.

### Sensitivity tests in duplex real-time PCR

**Fig. 1:**
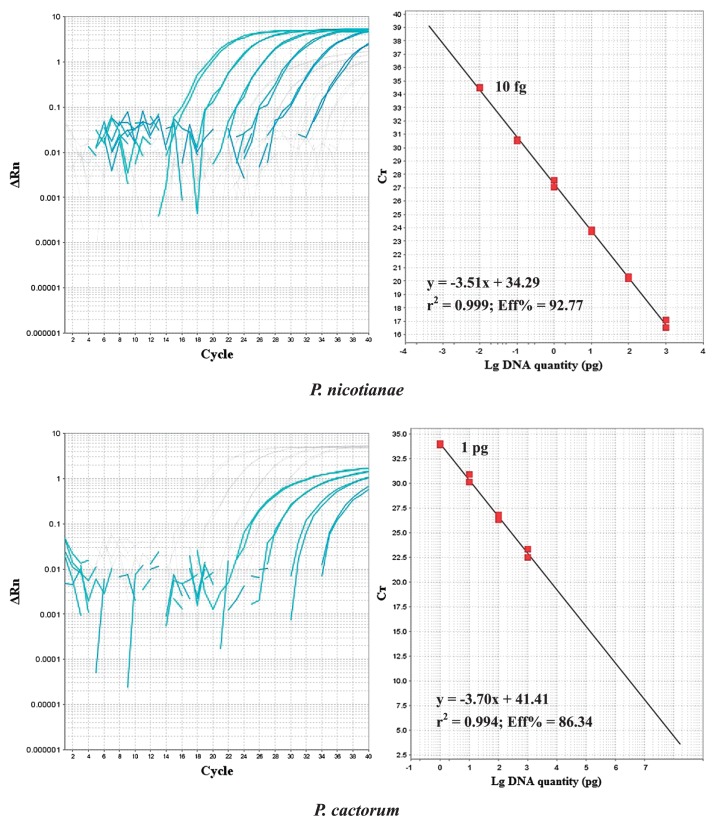
Detection limits, standard curves, correlation coefficients and amplification efficiencies assessed for *Phytophthora nicotianae* and *P. cactorum*. Total DNA from the two species was mixed together and serially diluted to yield final concentrations ranging from 1 ng μL^−1^ to 1 fg μL^−1^ before duplex real-time PCR amplification.

Sensitivities for *P. nicotianae* and *P. cactorum* DNA were tested. DNA from *P. nicotianae* isolate CH03OKTYPE3 and *P. cactorum* isolate GF654 were combined and then serially diluted from 1 ng μL^−1^ to 1 fg μL^−1^ before duplex real-time PCR. As a negative control, template DNA was replaced by sterilized distilled water. The detection limits of duplex real-time PCR were 10 fg target DNA for *P. nicotianae* and 1 pg for *P. cactorum* (Fig. 1). Standard curves showed a linear correlation between input DNA and cycle threshold (*C*_t_) values with correlation coefficients (*r*^2^) of 0.999 (*P. nicotianae*) and 0.994 (*P. cactorum*). The amplification efficiency for each target DNA was 92.77% (*P. nicotianae*) and 86.34% (*P. cactorum*), respectively. Analogous tests were also performed with DNA mixtures prepared from *P. nicotianae* isolate GF465 and *P. cactorum* isolate EID2. Identical detection limits were obtained. Additional tests of the detection limits for each species were executed using simplex real-time PCR. The same detection limits were obtained.

### Correlations between DNA quantities in diseased strawberry roots and the surrounding soils

Fifteen roots with surrounding soil, collected from diseased strawberry plants in the Saga strawberry greenhouse, were used to investigate the correlations between DNA quantities in roots and soils for *P. nicotianae* and *P. cactorum*. DNA extracts were analyzed by duplex real-time PCR for the quantification of *P. nicotianae* and *P. cactorum*. In 15 root samples, 9 samples showed the presence of *P. nicotianae* and *P. cacorum*, 4 samples showed only *P. nicotianae*, and the remaining 2 samples did not show any DNA of *Phytophthora*. The target DNA concentrations ranged from 25 to 83,844 pg (g root)^−1^ for *P. nicotianae* and from 8789 to 156,066 pg (g root)^−1^ for *P. cactorum*. Six of the 15 soil samples showed the presence of the two pathogens, and five other samples showed the presence of *P. nicotianae* only. No traces of the pathogens were found in the remaining four samples. The target DNA concentrations ranged from 16 to 19,627 pg (g soil)^−1^ for *P. nicotianae* and from 14 to 12,816 pg (g soil)^−1^ for *P. cactorum*.

**Fig. 2:**
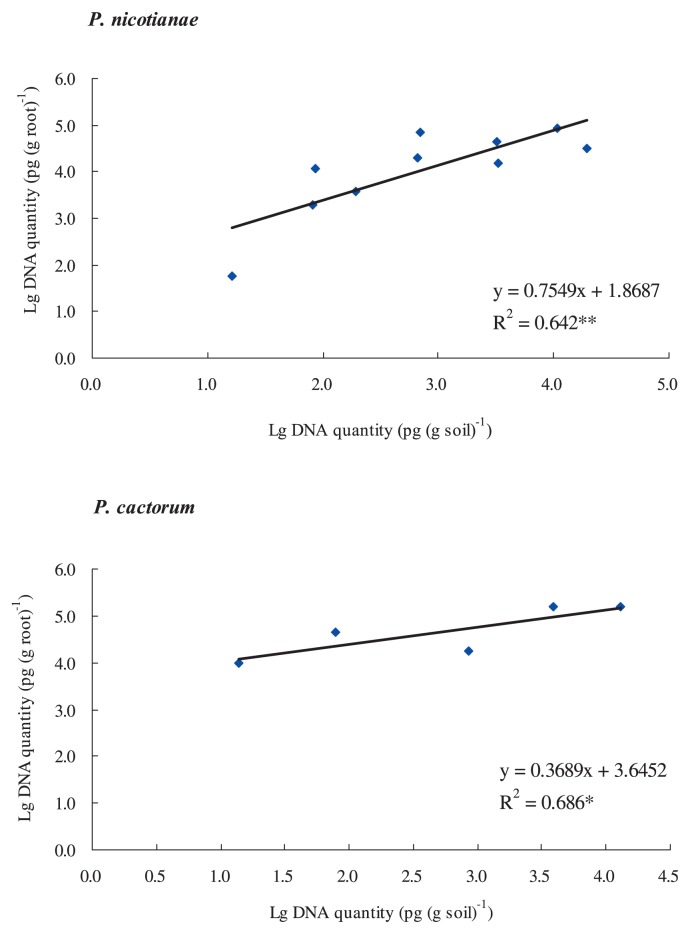
Correlations between DNA quantities in soils and roots for *P. nicotianae* and *P. cactorum*. Roots and soils surrounding the roots were collected from the diseased strawberry plants and the surrounding soils in a strawberry planting greenhouse. The DNA extracts were applied in the duplex real-time PCR for the quantifications of *P. nicotianae* and *P. cactorum*. Significance level: * = 5%, ** = 1%.

In those cases in which both root and soil were infested by the same pathogen, correlation analyses of the DNA quantities in root and soil were performed (Fig. 2). Linear correlations were found and the significance levels were 1% and 5% for *P. nicotianae* and *P. cactorum*, respectively (Fig. 2).

### Distribution of *P. nicotianae* and *P. cactorum* in strawberry greenhouses

**Fig. 3:**
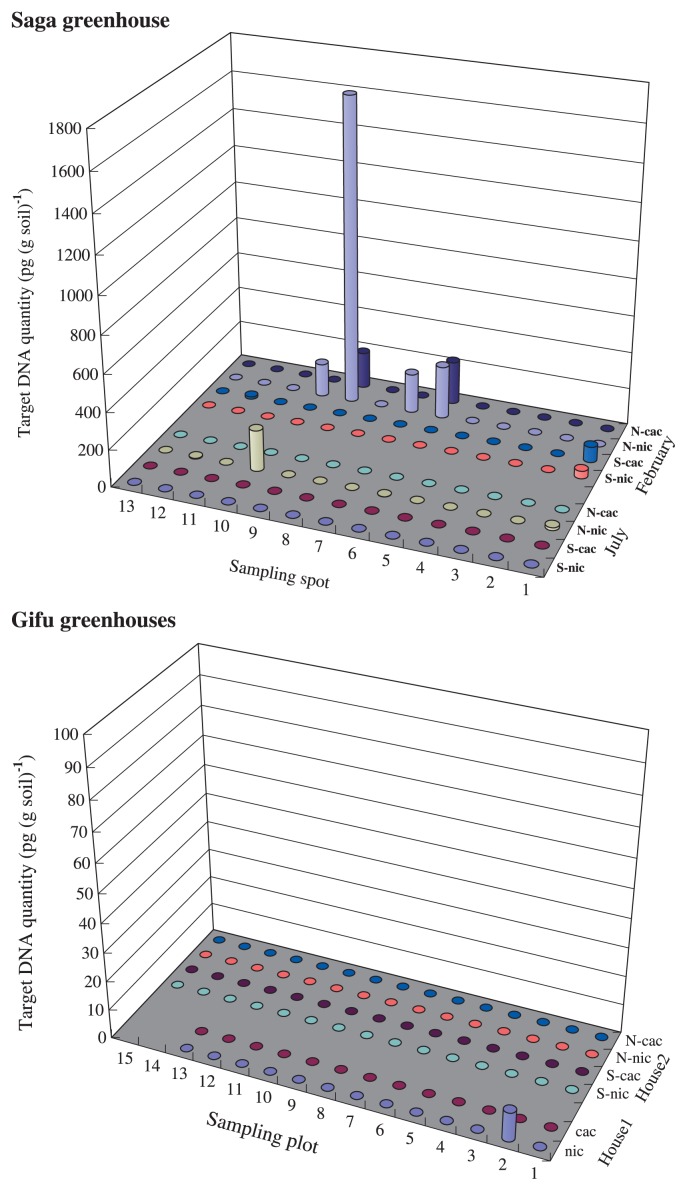
Distributions of *Phytophthora nicotianae* and *P. cactorum* in Saga and Gifu strawberry greenhouses. The soil DNA extracts were applied in duplex real-time PCR for the detection of *P. nicotianae* and *P. cactorum*. N = north side of strawberry greenhouse; S=south side; nic=*P. nicotianae*; cac=*P. cactorum*.

The distributions of *P. nicotianae* and *P. cactorum* in one strawberry greenhouse in Saga prefecture and two greenhouses in Gifu prefecture were investigated. In the Saga greenhouse, sampling was executed in July 2011 and February 2012. In July, *P. nicotianae* was detected in four samples (N1, N6, N10, and N12) with the target DNA quantity ranging from 1 to 221 pg (g soil)^−1^ (Fig. 3). *Phytophthora cactorum* was not detected. In February, three samples (S1, N6, and N9) were infested by both *P. nicotianae* and *P. cactorum*. In addition, two (N7, N10) and one (S12) of the plots were infested by *P. nicotianae* and *P. cactorum*, respectively. The target DNA concentrations ranged from 41 to 1,655 pg (g soil)^−1^ for *P. nicotianae* and from 13 to 233 pg (g soil)^−1^ for *P. cactorum* (Fig. 3). In the two Gifu greenhouses, *P. nicotianae* was detected in only one plot, with a DNA concentration of 10 pg (g soil)^−1^ (Fig. 3). *Phytophthora cactorum* was not found.

In the Saga greenhouse, we found symptoms of root rot in the strawberry plants in four plots, S1, N6, N7 and N9. No disease symptoms occurred in the plots (S12 and N10) where *P. cactorum* and *P. nicotianae* had been detected.

## Discussion

In this study, we developed a duplex real-time PCR technique to identify and quantify *P. nicotianae* and *P. cactorum* simultaneously. New species-specific primer pairs and TaqMan probes were designed for the ITS region of *P. nicotianae* and the *Ypt*1 gene of *P. cactorum*. The technique yielded an increase in fluorescence signals exclusively from the target species, but not from other *Phytophthora* species tested. Duplex real-time PCR was optimized and detection limits were determined using pure culture DNA. Using the optimized methodology, the distribution of the two pathogens in two strawberry planting areas of Japan was investigated.

The design of species-specific primers and probes for *P. nicotianae* and *P. cacorum* is critical, and the primers described in a previous report (24) had low specificity to *P. nicotianae* and *P. cactorum*. Although the specific primers designed by Li *et al.* (24) were competent for common multiplex PCR, they were not found suitable for multiplex real-time PCR due to difficulties in identifying an adequate probe. We attempted to set the Tm value of the primers as close as possible to each other. Because of poor inter-species variations in the ITS region and *Ypt*1 gene of *Phytophothora* Clade 1 species (3), the region available for primer design was limited; therefore, setting the Tm values of all four primers to 58°C was impossible; optimal values for the obtained specific primers for *P. nicotianae* and *P. cactorum* ranged from 53.7 to 58.8°C (Table 3). An optimal TaqMan probe-based real-time PCR should utilize probes with Tm values 10°C higher and amplicons of 50–150 bp. In this study, Tm values of TaqMan probes for *P. nicotianae* and *P. cacorum* were 67.2 and 63.2°C respectively, and were 9°C higher than those of the corresponding primers. Amplicon sizes of *P. nicotianae* and *P. cactorum* were 312 and 122 bp, respectively (Table 3).

Although ITS regions are widely used to identify and detect *Phytophthora* species, they are not always sufficiently diverse to allow the separation of closely related taxa. This was confirmed in the present study, although we successfully differentiated *P. nicotianae* from other *Phytophthora* species using ITS region primers; however, this region did not enable the differentiation of *P. cactorum* from other species. The ras-related protein gene *Ypt*1 seemed a more promising target as it provides sufficient variation to allow for the development of molecular markers for almost all *Phytophthora* species (31). Based on the *Ypt*1 gene, Schena *et al.* (30) designed *Phytophthora* genus-specific primers and specific primers for 15 *Phytophthora* species. These authors (31) also developed a multiplex real-time PCR for the detection and quantification of four *Phytophthora* species, including *P. ramorum*, *P. kernoviae*, *P. quercina* and *P. citricola*. We successfully designed primers and probes specific for *P. cactorum* using the *Ypt*1 gene.

Unlike rDNA genes which generally are present in multiple copies, the *Ypt*1 gene exists as a single copy only (6). In sensitivity tests, *P. cactorum* DNA was detected down to 1 pg, while the *P. nicotianae* DNA was detected down to 10 fg in simplex as well as duplex real-time PCR (Fig. 1). The different levels of sensitivity may be explained by the fact that rDNA genes occur in multiple copies (414±12 copies per haploid genome in *P. infestans*) (16). To improve the sensitivity for the *Ypt*1 gene, Schena *et al.* (30) used a nested approach based on first round amplification with *Phytophthora* genus-specific primers and a second amplification with species-specific multiplex real-time PCR. Although the sensitivity was increased to a level of 100 fg, it did not improve as greatly as expected. Nonetheless, this level of sensitivity appears sufficient for detection and quantification, indicating the potential of the nested PCR approach to improve sensitivity. For most practical applications, the lower level of sensitivity achieved with the *Ypt*1 gene might be a minor problem; however, the fact that the gene exists in a single copy suggests that single propagules of target species could be detected by a single multiplex real-time PCR. Methods based on single copy genes are not affected by the number of repeats as in multi-copy genes, and there is the potential to correlate *C*_t_ values accurately with the pathogen biomass and/or the number of propagules.

False negatives can occur in PCR-based detection methods; a variety of naturally occurring compounds, such as humic acids, tannins, and lignin-associated compounds can interfere with PCR reactions and inhibit amplification (4, 8). Therefore, prior assessment of DNA quality is essential despite recent improvements in DNA extraction procedures. Using the DNA extraction method refined by Kageyama *et al.* (17) and modified by incorporating a magnetic bead purification step (24), we ensured high quality and sufficient quantity of the extracted DNA, as further corroborated by pre-amplification with two 18S gene universal primers (1).

The soil dilution plating method is commonly used to estimate the quantity of fungi, based on selective culture media; however, the method cannot be used for the quantification of soil-borne pathogens, *P. nicotianae* and *P. cactorum*. The duplex real-time PCR developed in this study allows the simultaneous quantification of *P. nicotianae* and *P. cactorum* by detecting the concentration of target DNA. Using serial dilutions of this target DNA, linear responses and high correlation coefficients between the amount of DNA and the cycle thresholds were achieved. Target DNAs of *P. nicotianae* and *P. cactorum* in diseased strawberry roots and the surrounding soils were quantified, and significant correlations were found between DNA quantities in roots and soils (Fig. 2), which confirmed that high concentrations of pathogens in the soil possibly lead to a high risk of infection. In the soils around dead strawberry roots, we found DNA concentrations of *P. nicotianae* ranging from 16 to 19,627 pg (g soil)^−1^, and of *P. cactorum* ranging from 14 to 12,816 pg (g soil)^−1^. These results suggested that disease might develop when the DNA concentration of *P. nicotianae* or *P. cactorum* is more than 20 pg (g soil)^−1^.

In two of our 15 root samples, no pathogen was detected, possibly because the disease was anthracnose rather than *Phytophthora* rot, both of which produce similar symptoms. Regarding the lower incidence of pathogen detection in soils compared to roots, two possible explanations should be considered, namely extremely low pathogen populations and lower sensitivity for *P. cactorum*.

The distribution of *P. nicotianae* and *P. cactorum* in three strawberry greenhouses was determined using our new method. In one of 13 plots studied in Gifu greenhouse 1, only *P. nicotianae* was detected, while neither pathogen was detected in Gifu greenhouse 2. In the Saga greenhouse, *P. nicotianae* was detected in four of 26 plots in July and in five plots in February, while *P. cactorum* was not detected in July but was detected in four plots in February. Thus, the results of duplex real-time PCR showed that the distribution of *P. nicotianae* and *P. cacorum* in the greenhouses of strawberry would not be uniform.

Comparing DNA quantities between July 2011 and February 2012 in the Saga greenhouse, we concluded that the populations of *P. nicotianae* and *P. cactorum* had increased sharply. In addition, disease symptoms had occurred by February in the plots where *P. nicotianae* or *P. cactorum* were detected. The results suggest that soil sterilization was not sufficient to avoid an outbreak of the disease, and that the remaining pathogens would quickly propagate and affect the strawberry plants. In the S12 plot, real-time PCR showed the presence of *P. cactorum* but no symptom was found, probably because of the low pathogen density as indicated by the low DNA quantity of 13 pg (g soil)^−1^. This interpretation is consistent with the DNA concentration levels discussed above. An exception was found in plot N10. *Phytophthora nicotianae* was detected in July 2011 and February 2012, with DNA concentrations around 200 pg (g soil)^−1^; however, disease symptoms did not develop.

In conclusion, we described the first duplex real-time PCR method to simultaneously detect and quantify two important pathogens, *P. nicotianae* and *P. cactorum*. Based on this method, the distributions of the two pathogens in culturing fields could be known, and the occurrence of disease could be predicted. Our method proved to be rapid and reliable, and has great potential as a tool for identification and quantification in pathogen surveys and disease control.

## Figures and Tables

**Table 1:** Fungal species used in this study and their responses to PCR primers specific to *Phytophthora nicotianae* and *P. cactorum*.

Species	Isolate^a^	Host	Location	Its-nicF1/R3		Ypt-cacF3/R3	
	
				Conventional PCR	Real-time PCR	Conventional PCR	Real-time PCR
*P. nicotianae*	CH02FPK3	Strawberry	Chiba, Japan	+^b^	+^b^	−c	−c
	GF465	Strawberry	Gifu, Japan	+	+	−	−
	GF101	Karankoe	Gifu, Japan	+	N^d^	−	N^d^
	CBS305.29	Tobacco	Taiwan	+	N	−	N
	CBS101655	Alstromerea	Netherland	+	N	−	N
	C08	Ardisia crispa	Chiba, Japan	+	N	−	N
	CH03OKTYPE3	Strawberry	Okayama, Japan	+	+	−	−
*P. cactorum*	GF654	Strawberry	Gifu, Japan	−	−	+	+
	CH03OKTYPE1	Strawberry	Okayama, Japan	−	−	+	+
	CH99PFT4	Strawberry	Chiba, Japan	−	N	+	N
	CH01FPA1	Strawberry	Chiba, Japan	−	N	+	N
	CH07INBA1-2	Strawberry	Chiba, Japan	−	N	+	N
	CH02PMN001	Strawberry	Tokushima, Japan	−	N	+	N
	EID2	Strawberry	Chiba, Japan	−	−	+	+
*P. cajani*	WPC3105	*Cajanus cajan*	India	−	N	−	N
*P. cambivora*	WPC6358	Almond	Australia	−	N	−	N
*P. capsici*	WPC0253	Cacao	Mexico	−	N	−	N
*P. cinnamomi*	NBRC33182	*Hypericum androsaemum*	Japan	−	N	−	N
*P. citropthora*	WPC1200	*Cacao*	Brazil	−	N	−	N
*P. clandestina*	CBS347.86	*Trifolium subterraneum*	Australia	−	−	−	−
*P. erythroseptica*	WPC0340	Potato	Australia	−	N	−	N
*P. hedraiandra*	CBS111725	*Viburnum* sp.	Netherlands	−	−	−	−
*P. heveae*	WPC1102	Avocado	Guatemala	−	N	−	N
*P. humicola*	WPC3826	NA^e^	Taiwan	−	N	−	N
*P. idaei*	CBS971.95	*Rubus idaeus*	UK	−	−	−	−
*P. infestans*	CBS368.51	*Solanum tuberosum*	Netherlands	−	−	−	−
*P. insolita*	WPC6159	NA	NA	−	N	−	N
*P. ipomoeae*	CBS122203	Ingolfiella longipes	Mexico	−	−	−	−
*P. iranica*	CBS374.72	Solanum melongena	Iran	−	−	−	−
*P. meadii*	WPC3500	NA	NA	−	N	−	N
*P. medicaginis*	WPC7029	Alfalfa	USA	−	N	−	N
*P. megasperma*	WPC3163	NA	USA	−	N	−	N
*P. melonis*	WPC1371	Cucumber	NA	−	N	−	N
*P. mirabilis*	CBS678.85	*Mirabilis jalapa*	Mexico	−	−	−	−
*P. multivesiculata*	CBS545.96	*Cymbidium* sp.	Netherlands	−	−	−	−
*P. palmivora*	WPC0113	Papaya	USA	−	N	−	N
*P. phaseoli*	CBS120373	*Phaseolus lunatus*	USA	−	−	−	−
*P. pseudotsugae*	CBS444.84	*Pseudotsuga menziesii*	USA	−	−	−	−
*P. richardiae*	WPC7788	Carrot	United Kingdom	−	N	−	N
*P. sojae*	NBRC31016	*Glycine max*	Japan	−	N	−	N
*P. tentaculata*	C45	*Calendula arvensis*	Chiba, Japan	−	−	−	−
*P. undulate*	WPC7505	NA	NA	−	N	−	N
*P. vignae*	HoAz1	Azki bean	Hokkaido, Japan	−	N	−	N
*Plasmodiophora brassicae*	An	Chinese cabbage	Mie, Japan	−	N	−	N
*Pythium helicoides*	CBS286.31	*Phaseolus vulgaris*	USA	−	N	−	N
*Py. irregulare*	NBRC100108	Carrot	Gifu, Japan	−	N	−	N
*Py. myriotylum*	NBRC100113	Kidney bean	Hokkaido, Japan	−	N	−	N
*Py. ostracodes*	CBS768.73	Soil	Spain	−	N	−	N
*Py. paddicum*	NBRC31993	*Hordeum vulgare*	Japan	−	N	−	N
*Py. pyrilobum*	NBRC32560	*Agrostis palustris*	NA	−	N	−	N
*Py. spinosum*	NBRC100116	Soil	Gifu, Japan	−	N	−	N
*Py. sylvaticum*	NBRC100119	Soil	Gifu, Japan	−	N	−	N
*Py. ultimum*	NBRC100123	Soil	Gifu, Japan	−	N	−	N
*Py. vexans*	MS6-10-8V	Soil	Gifu, Japan	−	N	−	N
*Pyrenochaeta lycopercisi*	Type1	Tomato	Japan	−	N	−	N
*Rhizoctonia solani*	RGR38	NA	Japan	−	N	−	N
*Saprolegnia* sp.	NBRC32708	*Salmo trutta*	NA	−	N	−	N
*Verticillium albo-atrum*	Vaal 130308	NA	NA	−	N	−	N
*V. dahliae*	Vd84034	NA	NA	−	N	−	N

a Isolates were collected from CBS (Centraalbureau voor Schimmelcultures), NBRC (NITE Biological Research Centre), WPC (World *Phytophthora* Genetic Resource Collection) and Gifu University Cultures Collection.

b Amplified.

c No amplification.

d Not tested.

e Not accessible.

**Table 2:** Accession numbers of ITS region and *Ypt*1 gene sequences of *Phytophthora* species in GenBank DNA database

Species	ITS region		*Ypt*1 gene	
	
	Isolates	Accession	Isolates	Accession
*P. nicotianae*	P1452	FJ801769	IMI268688	DQ162981
	P7146	FJ801963	CH02FPK3	HQ849999
	P11000	FJ801542		
*P. cactorum*	CH98PEC1	AB367364	IMI296524	DQ162960
	CH03OKTYPE1	AB367366	CH03OKTYPE1	HQ850000
	CH02MKPY001	AB367365	EID2	HQ850001
*P. alni* subsp. *alni*	P16203	GU259292	SCRP2	DQ162953
*P. bisheria*	Cg.2.3.3	AY241924	N^a^	N^a^
*P. botryosa*	P6945	FJ801954	N	N
*P. cambivora*	P0592	GU259025	SCRP82	DQ162956
*P. capsici*	P1091	GU259193	IMI352321	DQ162972
*P. chrysanthemi*	GF749	AB437135	N	N
*P. cinnamomi*	P3232	GU594781	CBS270.55	DQ162959
*P. citricola*	P7902	GU259136	SCRP143	DQ162971
*P. citrophthora*	P6310	FJ801913	IMI332632	DQ162973
*P. clandestina*	P3942	FJ801888	CBS347.86	HQ850002
*P. colocasiae*	P6318	GU258989	N	N
*P. cryptogea*	CBS290.35	AF228099	IMI045168	DQ162987
*P. drechsleri*	P10331	FJ801387	ATCC46724	DQ162989
*P. erythroseptica*	CBS 956.87	AF228082	SCRP240	DQ162988
*P. europaea*	CBS109049	DQ275190	SCRP622	DQ162952
*P. fragariae* var. *fragariae*	AF266762	SCRP245	DQ162950	
*P. hedraiandra*	P11056	EU080072	CBS111725	HQ850003
*P. idaei*	P6767	FJ801946	CBS971.95	HQ850004
*P. ilicis*	P2159	AY302164	SCRP379	DQ162963
*P. infestans*	P10650	FJ801470	CBS368.51	HQ850005
*P. inflate*	IMI342898	AF266789	N	N
*P. insolita*	IMI288805	AF271222	IMI288805	DQ162974
*P. inundata*	P8478	FJ802005	SCRP649	DQ162985
*P. ipomoeae*	P10225	FJ801323	CBS122203	HQ850006
*P. iranica*	CBS374.72	L41378	CBS374.72	HQ850007
*P. katsurae*	P10187	GU259517	SCRP388	DQ162980
*P. kernoviae*	P1571	AY940661	SCRP722	DQ162975
*P. lateralis*	P3888	FJ802093	IMI040503	DQ162991
*P. meadii*	P6128	GU259180	N	N
*P. medicaginis*	P10683	GU259090	SCRP407	DQ162990
*P. megakarya*	P8516	FJ802010	P8517	HQ850008
*P. megasperma*	P3136	GU258789	IMI133317	DQ162986
*P. melonis*	P10994	FJ801540	PMNJHG1	EF649778
*P. mexicana*	P0646	FJ801253	N	N
*P. mirabilis*	P3005	FJ802098	CBS678.85	HQ850009
*P. multivesiculata*	CBS545.96	DQ988192	CBS545.96	HQ850010
*P. nemorosa*	P10288	FJ801359	SCRP910	DQ162965
*P. palmivora*	P0255	FJ801246	IPPc3	HQ850011
*P. parsiana*	C25	AY659739	N	N
*P. phaseoli*	P10145	FJ802106	CBS120373	HQ850012
*P. pistaciae*	P6197	FJ801904	IMI386658	DQ162957
*P. polonica*	P131445	AB511828	N	N
*P. pseudosyringae*	P10437	FJ801438	SCRP734	DQ162967
*P. pseudotsugae*	IMI331662	AF266774	CBS444.84	HQ850013
*P. psychrophila*	P10433	FJ801435	SCRP630	DQ162964
*P. quercina*	CBS 115973	AY853200	SCRP550	DQ162979
*P. ramorum*	P10301	FJ801362	SCRP911	DQ162992
*P. richardiae*	RICH-P7789	AB367498	N	N
*P. sojae*	P3114	FJ801828	SCRP555	DQ162958
*P. tentaculata*	CBS552.96	AF266775	C45	HQ850014
*Pythium oedochilum*	N	N	CBS597.68	HQ850015
*Py. helicoides*	H5sz1C14	AB108025	TCG3	HQ850016
*Py. ostracodes*	N	N	CBS768.73	HQ850017
*Py. undulatum*		AF271230	N	N
*Py. vexans*	CBS 119.80	AY598713	N	N

a Species not used for this DNA region.

**Table 3:** PCR primers and TaqMan probes designed in this study

Primers/Probes	Sequences (5′→3′)	Length (bp)	Tm (°C)^e^	Amplicon size (bp)
Its-nicF1^a^	CCTATCAAAAAAAAGGCGAACG	22	58.8	
Its-nicR3^a^	TACACGGAAGGAAGAAAGTCAAG	23	56.4	312
P-nic4^b^	CGGACACTGATACAGGCATACTTCCAGG	28	67.2	

Ypt-cacF3^c^	CATGGCATTATCGTGGTGTA	20	54.0	
Ypt-cacR3^c^	GCTCTTTTCCGTCGGC	16	53.7	122
P-cac4^d^	CGGACCAGGAGTCGTTCAACAAC	23	63.7	

a Specific primers for *P. nicotianae*.

b TaqMan probe for *P. nicotianae*.

c Specific primers for *P. cactorum*.

d TaqMan probe for *P. cactorum*.

e Tm values are calculated using the nearest-neighbor algorithm.
